# Convolutional neural network -based phantom image scoring for mammography quality control

**DOI:** 10.1186/s12880-022-00944-w

**Published:** 2022-12-07

**Authors:** Veli-Matti Sundell, Teemu Mäkelä, Anne-Mari Vitikainen, Touko Kaasalainen

**Affiliations:** 1 Department of Physics, University of Helsinki, P.O. Box 64, 00014 Helsinki, Finland; 2 HUS Diagnostic Center, Radiology, University of Helsinki and Helsinki University Hospital, P.O. Box 340, Haartmaninkatu 4, 00290 Helsinki, Finland

**Keywords:** Mammography, Quality control, Convolutional neural network

## Abstract

**Background:**

Visual evaluation of phantom images is an important, but time-consuming part of mammography quality control (QC). Consistent scoring of phantom images over the device’s lifetime is highly desirable. Recently, convolutional neural networks (CNNs) have been applied to a wide range of image classification problems, performing with a high accuracy. The purpose of this study was to automate mammography QC phantom scoring task by training CNN models to mimic a human reviewer.

**Methods:**

Eight CNN variations consisting of three to ten convolutional layers were trained for detecting targets (fibres, microcalcifications and masses) in American College of Radiology (ACR) accreditation phantom images and the results were compared with human scoring. Regular and artificially degraded/improved QC phantom images from eight mammography devices were visually evaluated by one reviewer. These images were used in training the CNN models. A separate test set consisted of daily QC images from the eight devices and separately acquired images with varying dose levels. These were scored by four reviewers and considered the ground truth for CNN performance testing.

**Results:**

Although hyper-parameter search space was limited, an optimal network depth after which additional layers resulted in decreased accuracy was identified. The highest scoring accuracy (95%) was achieved with the CNN consisting of six convolutional layers. The highest deviation between the CNN and the reviewers was found at lowest dose levels. No significant difference emerged between the visual reviews and CNN results except in case of smallest masses.

**Conclusion:**

A CNN-based automatic mammography QC phantom scoring system can score phantom images in a good agreement with human reviewers, and can therefore be of benefit in mammography QC.

## Introduction

Conventional mammography, a two-dimensional X-ray imaging modality for detecting and diagnosing certain breast diseases, is widely applied in breast cancer screening. To ensure adequate image quality and appropriate radiation dose level for all patients, systematic quality assurance is needed and often is mandatory. An essential part of technical mammography quality control (QC) is a periodic imaging of a test object. These phantoms usually contain targets mimicking fibrous structures (fibres), microcalcifications, and tumorous masses, simulating relevant imaging findings. The visibility of these targets is assessed from the acquired phantom images. This has traditionally been done by human reviewers. Alternatively, a dedicated software may be used. Time savings, objectivity, and repeatability (over long periods of time) are prime motivations for software automation.

Different kind of model observers have been used for automatic image quality evaluations [1]. These models aim to predict human performance in specific tasks. The models include e.g. ideal Bayesian, non-prewhitening matched filter with an eye filter, channelized ideal and channelized Hotelling observers [1]. Model observers have been used in wide variety of image quality evaluations, including mammography and digital breast tomosynthesis (DBT) [2–5].

Recently, convolutional neural networks, CNNs, have outperformed traditional image processing algorithms in a multitude of challenging classification tasks [6], at times reaching or even exceeding human performance [7]. A neural network consists of input, hidden, and output layers with non-linear activation functions and trainable weights between neurons. In supervised learning, the goal is to map the input to a desired (ground truth) output. CNN is a special case of neural networks utilizing spatial filters, the values of which are determined during the iterative learning process. Convolutional layers maintain spatial relationships and considerably reduce the required number of parameters compared, for example, with fully connected layers. This makes them feasible for image processing applications. Network architecture choices including network depth, the size and number of filters, pooling and normalization layers, activation functions, and training parameters constitute a large hyper-parameter space that remains to be chosen by the data scientist. Increasing the complexity and the number of trainable parameters increases the model’s representative power. However, it may hinder training and may encourage overfitting, i.e. poor generalization to unseen images. In general, increasing the size of the training dataset tends to improve the network performance.

CNNs have shown great promise in many radiological applications such as computer-aided detection [8], image segmentation [9, 10], and image reconstruction [11–13]. CNNs have been used for breast tissue classification [14], breast density segmentation, risk scoring [15, 16], mass and microcalcification detection [17–21] in both conventional mammography and in DBT. Additionally, CNN-based observers have successfully been used in image quality evaluation for mammography and DBT [22–24].

Traditional visual scoring of QC phantom images is essentially a set of binary classification tasks. For example, the American College of Radiology (ACR) instructs the reviewer to assess the visibility (visible or not) of each fibre, microcalcification group, and mass in the accreditation phantom mammograms. Also, the respective acceptance criteria are provided. Not all targets are required to be visible with typical imaging parameters, and superfluous detectability could indicate unjustified dose. In earlier studies, Fourier transform [25], cross-correlation coefficients between observed and reference images [26, 27], Mahalanobis distance-based methods [26], and the discrete wavelet transform (DWT) [28, 29] have been used for automating image evaluation of the ACR phantom.

The aim of this study was to automate mammography QC phantom scoring task by training CNN models to mimic a human reviewer. The training dataset was constructed from archived daily QC images with and without noise augmentation. The final model performance was evaluated on a test set comprising of standard QC phantom images and images with levels of noise not seen in the standard QC images.

## Materials and methods

### Image acquisition

The ACR accreditation phantoms are imaged prior to the first patient of the day on all mammography systems in the Radiology department of HUS Diagnostic Center. Phantom placement and imaging are standardized. Automatic exposure control (AEC) and compression paddle are used. The QC images are reviewed both visually and sent to an image processing server for storage and complementary automatic analysis.

In this study, images from eight mammography systems with their respective ACR mammography phantoms were used. Seven mammography systems were full-field digital mammography (FFDM) systems and one a computed radiography (CR) mammography system. FFDM systems were from three vendors: three systems were from GE Healthcare (Waukesha, WI, USA), two systems from Siemens Healthcare (Erlangen, Germany), and two systems from Planmed (Helsinki, Finland). The CR mammography system was manufactured by Planmed with a Fujifilm (Tokyo, Japan) CR system. The image pixel size varied from 83 to 100 μm for the FFDM devices. The pixel size for the CR system was 50 μm. The image presentation intent type was ‘for presentation’ except for the Planmed FFDM systems for which ‘for processing’ was used. The ACR phantoms were produced by Gammex (Middleton, WI, USA) and CIRS (Computerized Imaging Reference Systems, Inc., Norfolk, VA, USA). The ACR mammography phantoms consist of six fibres of different diameters, five groups of microcalcifications of different sizes, and five masses of different thicknesses and diameters. All the targets are embedded in a wax block [30]. MATLAB (MathWorks, Natick, MA, USA) with Image Processing and Deep Learning Toolboxes was used for all image processing (versions R2018b and R2019b) and CNN training (version R2018b). In addition to the standard QC acquisitions, separate image sets with a wide range of dose levels were acquired on multiple devices.

### Image pre-processing

**Fig. 1:**
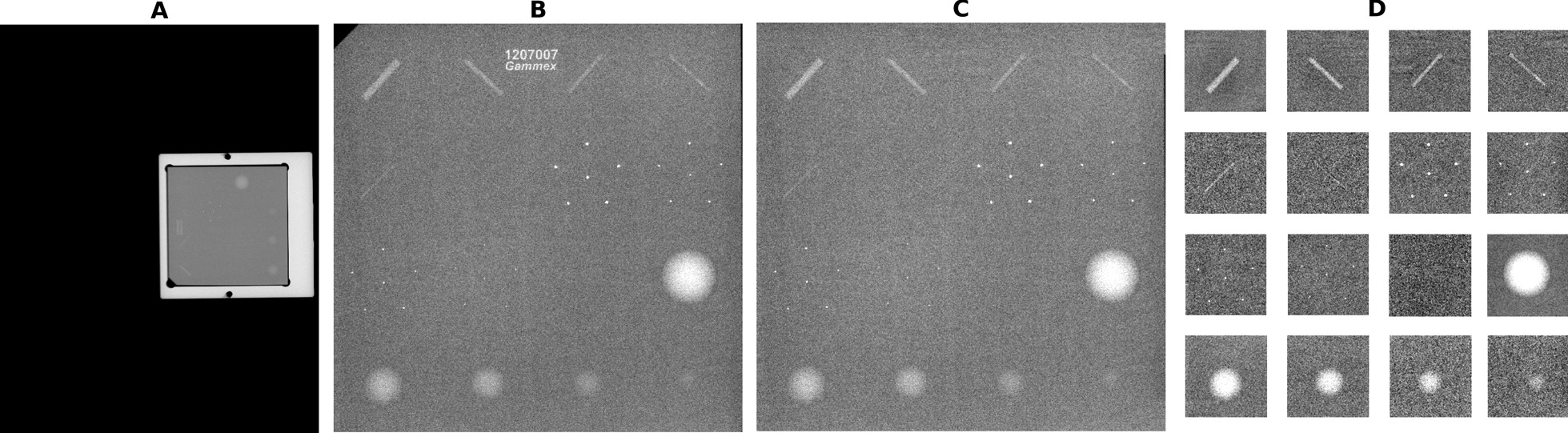
Pre-processing a phantom image (**A**) consisted of cropping and rotation of the wax area (**B**) and replacing the phantom name and corners with noise (**C**). Each fibre, mass, and microcalcification group was then separated into an individual sub-image (**D**)

The images were pre-processed to normalize the CNN inputs (see Fig. 1). The pre-processing was fully automatic and did not require user interaction. The phantom wax area was cropped from the image and rotation corrected when needed. In the wax area cropping process, the phantom was first extracted from the whole image based on its signal intensity which differed notably from the background. Secondly, the wax area was cropped from the phantom image by detecting the strong intensity changes along x- and y-axis. Finally, if needed, the wax area was rotated to the right orientation based on the location of the largest mass target. This method resulted in a minor variation in extracted wax area size, and 95% of the images per device were within 1% of the mean wax area size. The phantom model’s name visible in the images was replaced with noise to discourage the CNN from learning irrelevant features. The wax area image was divided into 16 square sub-images based on manually defined locations of the targets-of-interest. Each of these sub-images contained an individual fibre, mass, or microcalcification group (regardless of the target visibility). The sub-images were from 190 to 390 pixels wide depending mostly on the pixel sizes and to a lesser extent on the variations in the wax area cropping. From that point on, the sub-images were processed individually. Low-frequency background intensity non-uniformity was removed by subtracting two-dimensional polynomial fits from the sub-images. Fifth order polynomial was used for fibres and first order polynomial for microcalcifications and masses. The higher order was chosen for the fibres as they are thin low-contrast targets mostly located near the phantom edges, and thus more susceptible to background intensity non-uniformities. Finally, the sub-images were resized to 128 × 128 pixels, the intensities scaled to 0–1 range and stored as 64-bit double-precision floating-point values.

### Training and test datasets

Training dataset was composed with unmodified and modified QC images. Archived daily QC images routinely acquired by the sites’ radiographers were used for training dataset. The imaging conditions varied primarily due to slight differences in the phantom positioning on the breast support table and the resulting changes in the AEC values. The mean glandular doses (MGDs) were typically between 0.8 and 1.2 mGy. Because the original images had target conspicuity and noise levels close to each other, additional modified images were calculated by either reducing or increasing the image noise.

Artificially degraded images were calculated by adding realistic noise to the original QC phantom images. First, an individual noise template was created for each device. This template was created from regular QC phantom image by cropping a region in the phantom wax area that did not contain any targets. When creating degraded images, noise template was divided into 10 × 10-pixel patches that were randomly sampled. The final modified image was produced by weighted arithmetic mean between a random image from the system and a scaled randomly sampled noise template. Prior to the averaging, the noise template was scaled to have a mean value (in the wax area) equal to the original image. Noise mixing values from 30 to 80% with 5% steps were used. Ten images per the 11 noise levels per mammography system were generated, yielding 110 images per mammography system with increased noise levels.

The noise-reduced images were created by averaging regular QC phantom images together. The image alignment was performed semi-iteratively with 0.05° steps. The intermediate images during the iterative process (as well as the final images) were always created by rotating the original image by the current angle, i.e. without multiple interpolations. The rotation was based on detecting specific predefined microcalcifications by minimizing their reference and rotated locations. The number of averaged images were 2, 3, 4 and a varying number (from 5 to 20 depending on system performance) required to make all the phantom targets clearly visible. For each device, 30 images per averaging level were generated. This yielded 120 images per mammography system with reduced noise levels.

In total, 800 regular and 1840 modified phantom images were used in the training and validation dataset. Train-validation split was 90:10. A single reviewer (VMS) scored the training images.

The test data comprised of three subsets of images:

1. Ten regular QC phantom images from each of the eight mammography systems.
2. Ten different exposures ranging from 4 to 320 mAs, or approximately − 90% to + 490% of the standard QC levels, from three systems (GE, Siemens, and Planmed). Each exposure setting was imaged twice.
3. Five X-ray tube voltages (27–32 kVp) with low exposure values (6.3 mAs to 10.0 mAs) from two systems (GE and Planmed). Each voltage setting was imaged twice.

The MGDs ranged from 0.1 to 5.3 mGy. The second and third subsets were acquired to emulate changes in the systems’ performances. Four reviewers (the authors) scored all 160 images. All reviewers had earlier experience on medical physics and mammography. Reviewers 1 to 4 (as reported in the results) had 5 years, 13 years, 15 years and 9 years of experience in medical physics on the time of image review.

**Fig. 2:**
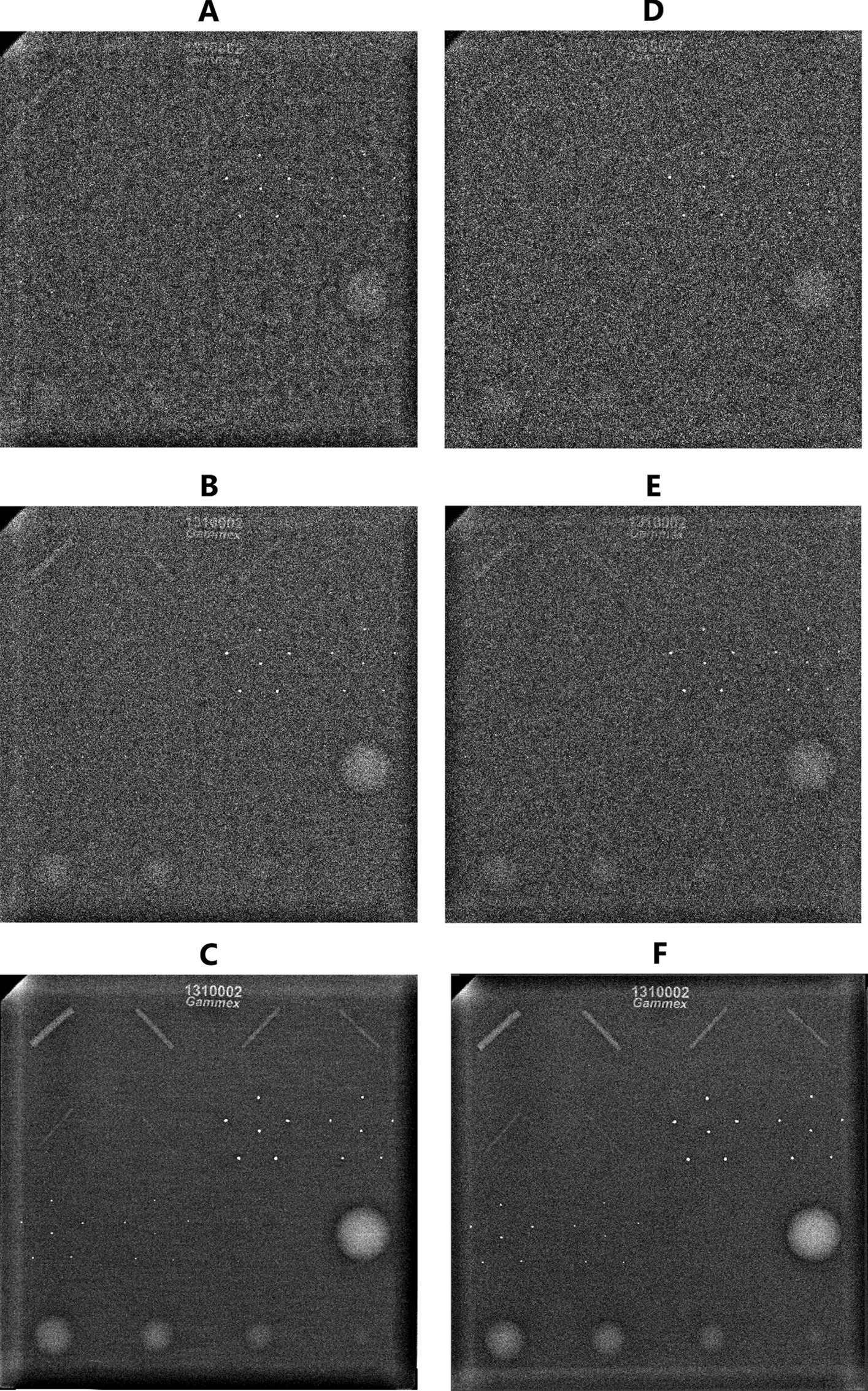
Unmodified (left column) test set images and modified train set images (right column) acquired with Siemens system. The ACR phantom consist of six fibres, five microcalcification groups, and five masses. **A**, **B**, and **C** show images acquired at 0.18 mGy, 0.31 mGy, and 4.00 mGy mean glandular doses, respectively. **D** and **E** represent degraded images with 60% and 45% noise mixing levels. F represents an average image of five ordinary QC control images. The visibility scores given for **A** and **D** were following: 1 point for fibres, 3 points for microcalcifications, and 1 point for masses (**A**) and 2.5 points for masses (**D**). The visibility scores given for **B** and **E** were following: 3 points for fibres, 4 points for microcalcifications, and 3.5 points for masses. The visibility scores for **C** and **F** were 6 points for fibres, 5 points for microcalcifications, and 5 points for masses. The visibility scores were taken from a single reviewer (VMS) scorings

**Fig. 3:**
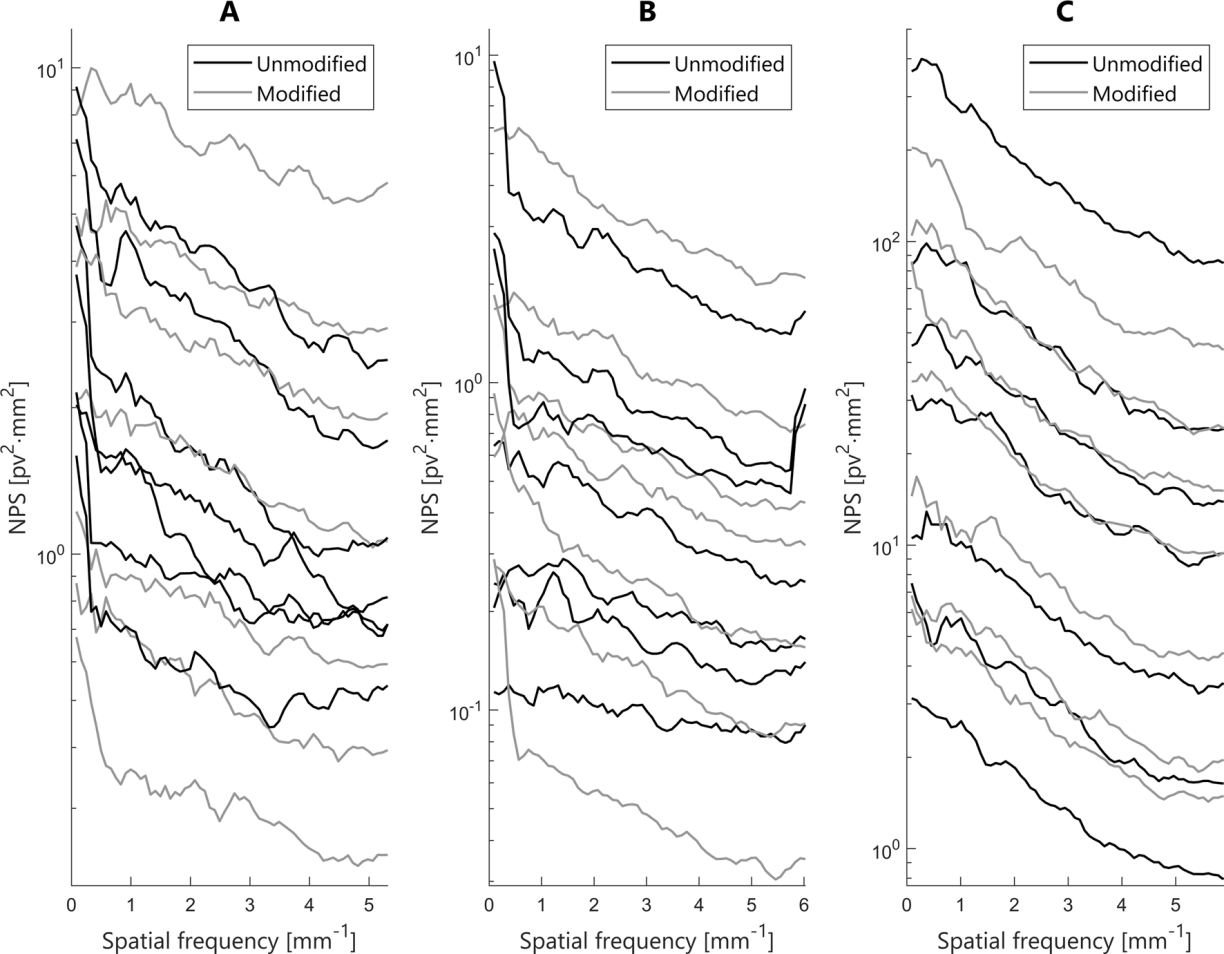
A selection of noise power spectrums of unmodified test and modified training images for GE (**A**), Planmed (**B**), and Siemens (**C**) systems. NPS curves of the four artificially degraded and three noise-reduced images are shown for each system (gray). NPS of four lower than regular QC exposures and three higher exposures are shown in black. The uppermost NPS curves with the highest noise content represent modified images with the highest noise mixing values and unmodified images with the lowest exposure values. Likewise, the lowermost NPS curves correspond to the highest number of image averages for modified images and the highest exposure for the unmodified images. Pv denotes pixel value and mm denotes millimetres. The curves were smoothed with 5-point moving average

The similarity between the unmodified test set images and the modified training images is presented in Fig. 2. Noise power spectrums (NPS) were calculated to demonstrate the comparability between the real acquisitions and the modified images (Fig. 3). The modified images for GE and Planmed systems covered the absolute noise power range of the unmodified images. However, the NPS curve shapes had slight differences. For Siemens system, the absolute noise power levels of the unmodified images were not fully covered by modified images compared to the unmodified images. However, the NPS curve shapes were more similar. The training set consisting of standard QC images but with artificial degradation and improvements was considered to cover the test set domain sufficiently, and no further evaluations on different augmentation tactics were performed.

### Visual scoring

The training data were scored using a pair of 3 MP grayscale displays (Barco MDCG-3120-CB, Barco NV, Kortrijk, Belgium). The test data were scored using 3 MP color displays (Barco MDNC-3421). DICOM compliance verifications [31] were performed for both monitor pairs. During the scoring process, 1:1 zoom was used (one pixel on the image equals one pixel on the monitor), and windowing was freely adjustable. The human observers viewed the full phantom images and scored the visibility of each individual fibre, microcalcification, and mass. The phantom image scoring followed the ACR guidelines for each separate target [30]. For fibres and masses, zero points was given to a non-visible target, half a point if it was barely visible, and one point to a visible target. Microcalcification group scoring was performed by scoring each individual microcalcification speck as either visible or not visible. Total number of visible microcalcifications was calculated for each group. If less than three microcalcifications were visible, the group was given zero points, three visible per group a half point, and more than three visible per group one point. For the training data, the single-reviewer scores were rounded directly to be either zero (for non-visible) or one (for visible and barely visible). For the test data, consensus scores were obtained for each target by taking the median of the reviewers’ scores and then rounding to either zero or one.

### Convolutional neural networks

Table 1 shows the details of the eight trained CNN models. CNN models showed in this study were chosen by using a trial-and-error method. The number of convolutional layers varied from three to ten. The 128 × 128 image input layer was followed by blocks of convolutional layer, batch normalization, rectified linear unit activation, max pooling, and dropout. These were followed by flattening, a fully connected layer, and a classification layer with softmax activation. The first convolution layer filter size was 5 × 5, and the rest were 3 × 3 with stride one in all. Max pooling layers had pool size of 3 × 3 and stride 2. For dropouts, 30% probability was used in between convolutional layers and 50% probability was used prior to the fully connected layer. The classification layers had 17 neurons; each visible target type had its own category (Fig. 1D), and one category was for the target being non-visible (regardless of which sub-image was in question). In effect, the model predicts from the input sub-image the visibility and identifies the target in question. This is a novel use of CNNs in mammography QC.

**Table 1:** Convolutional neural network architectures

CNN1 tp: 267,122	CNN2 tp: 386,902	CNN3 tp:236,662	CNN4 tp: 280,082	CNN5 tp: 434,762	CNN6 tp: 748,702	CNN7 tp: 1,025,102	CNN8 tp: 1,366,362
input	input	input	input	input	input	input	input
128 × 128, 5 × 5 conv, 30	128 × 128, 5 × 5 conv, 30	128 × 128, 5 × 5 conv, 30	128 × 128, 5 × 5 conv, 30	128 × 128, 5 × 5 conv, 30	128 × 128, 5 × 5 conv, 30	128 × 128, 5 × 5 conv, 30	128 × 128, 5 × 5 conv, 30
128 × 128 × 30, N + A + P	128 × 128 × 30, N + A + P	128 × 128 × 30, N + A + P	128 × 128 × 30, N + A + P	128 × 128 × 30, N + A + P	128 × 128 × 30, N + A + P	128 × 128 × 30 N + A + P	128 × 128 × 30, N + A + P
63 × 63 × 30, 3 × 3 conv, 45	63 × 63 × 30, 3 × 3 conv, 45	63 × 63 × 30, 3 × 3 conv, 45	63 × 63 × 30, 3 × 3 conv, 45	63 × 63 × 30, 3 × 3 conv, 45	63 × 63 × 30, 3 × 3 conv, 45	63 × 63 × 30, 3 × 3 conv, 45	63 × 63 × 30, 3 × 3 conv, 45
63 × 63 × 45, N + A + P	63 × 63 × 45, N + A + P	63 × 63 × 45, N + A + P	63 × 63 × 45, N + A + P	63 × 63 × 45, N + A + P	63 × 63 × 45, N + A + P	63 × 63 × 45, N + A + P	63 × 63 × 45, N + A + P
31 × 31 × 45, dropout, 30%	31 × 31 × 45, dropout, 30%	31 × 31 × 45, dropout, 30%	31 × 31 × 45, dropout, 30%	31 × 31 × 45, dropout, 30%	31 × 31 × 45, dropout, 30%	31 × 31 × 45, dropout, 30%	31 × 31 × 45, dropout, 30%
31 × 31 × 45, 3 × 3 conv, 60	31 × 31 × 45, 3 × 3 conv, 60	31 × 31 × 45, 3 × 3 conv, 60	31 × 31 × 45, 3 × 3 conv, 60	31 × 31 × 45, 3 × 3 conv, 60	31 × 31 × 45, 3 × 3 conv, 60	31 × 31 × 45, 3 × 3 conv, 60	31 × 31 × 45, 3 × 3 conv, 60
31 × 31 × 60, N + A + P	31 × 31 × 60, N + A + P	31 × 31 × 60, N + A + P	31 × 31 × 60, N + A + P	31 × 31 × 60, N + A + P	31 × 31 × 60, N + A + P	31 × 31 × 60, N + A + P	31 × 31 × 60, N + A + P
15 × 15 × 60, dropout, 50%	15 × 15 × 60, dropout, 30%	15 × 15 × 60, dropout, 30%	15 × 15 × 60, dropout, 30%	15 × 15 × 60, dropout, 30%	15 × 15 × 60, dropout, 30%	15 × 15 × 60, dropout, 30%	15 × 15 × 60, dropout, 30%
15 × 15 × 60, fully connected	15 × 15 × 60, 3 × 3 conv, 80	15 × 15 × 60, 3 × 3 conv, 80	15 × 15 × 60, 3 × 3 conv, 80	15 × 15 × 60, 3 × 3 conv, 80	15 × 15 × 60, 3 × 3 conv, 80	15 × 15 × 60, 3 × 3 conv, 80	15 × 15 × 60, 3 × 3 conv, 80
1 × 1 × 17, softmax	15 × 15 × 80, N + A	15 × 15 × 80, N + A + P	15 × 15 × 80, N + A + P	15 × 15 × 80, N + A + P	15 × 15 × 80, N + A + P	15 × 15 × 80, N + A + P	15 × 15 × 80, N + A + P
	15 × 15 × 80, dropout, 50%	7 × 7 × 80, dropout, 30%	7 × 7 × 80, dropout, 30%	7 × 7 × 80, dropout, 30%	7 × 7 × 80, dropout, 30%	7 × 7 × 80, dropout, 30%	7 × 7 × 80, dropout, 30%
	15 × 15 × 80, fully connected	7 × 7 × 80, 3 × 3 conv, 100	7 × 7 × 80, 3 × 3 conv, 100	7 × 7 × 80, 3 × 3 conv, 100	7 × 7 × 80, 3 × 3 conv, 100	7 × 7 × 80, 3 × 3 conv, 100	7 × 7 × 80, 3 × 3 conv, 100
	1 × 1 × 17, softmax	7 × 7 × 100, N + A	7 × 7 × 100, N + A + P	7 × 7 × 100, N + A + P	7 × 7 × 100, N + A	7 × 7 × 100, N + A	7 × 7 × 100, N + A
		7 × 7 × 100, dropout, 50%	3 × 3 × 100, dropout, 30%	3 × 3 × 100, dropout, 30%	7 × 7 × 100, dropout, 30%	7 × 7 × 100, dropout, 30%	7 × 7 × 100, dropout, 30%
		7 × 7 × 100, fully connected	3 × 3 × 100, 3 × 3 conv, 120	3 × 3 × 100, 3 × 3 conv, 120	7 × 7 × 100, 3 × 3 conv, 120	7 × 7 × 100, 3 × 3 conv, 120	7 × 7 × 100, 3 × 3 conv, 120
		1 × 1 × 17, softmax	3 × 3 × 120, N + A	3 × 3 × 120, N + A	7 × 7 × 120, N + A	7 × 7 × 120, N + A	7 × 7 × 120, N + A
			3 × 3 × 120, dropout, 50%	3 × 3 × 120, dropout, 30%	7 × 7 × 120, dropout, 30%	7 × 7 × 120, dropout, 30%	7 × 7 × 120, dropout, 30%
			3 × 3 × 120, fully connected	3 × 3 × 120, 3 × 3 conv, 140	7 × 7 × 120, 3 × 3 conv, 140	7 × 7 × 120, 3 × 3 conv, 140	7 × 7 × 120, 3 × 3 conv, 140
			1 × 1 × 17 softmax	3 × 3 × 140, N + A	7 × 7 × 140, N + A	7 × 7 × 140, N + A	7 × 7 × 140, N + A
				3 × 3 × 140, dropout, 50%	7 × 7 × 140, dropout, 30%	7 × 7 × 140, dropout, 30%	7 × 7 × 140, dropout, 30%
				3 × 3 × 140, fully connected	7 × 7 × 140, 3 × 3 conv, 160	7 × 7 × 140, 3 × 3 conv, 160	7 × 7 × 140, 3 × 3 conv, 160
				1 × 1 × 17, softmax	7 × 7 × 160, N + A	7 × 7 × 160, N + A	7 × 7 × 160, N + A
					7 × 7 × 160, dropout, 50%	7 × 7 × 160, dropout, 30%	7 × 7 × 160, dropout, 30%
					7 × 7 × 160, fully connected	7 × 7 × 160, 3 × 3 conv, 180	7 × 7 × 160, 3 × 3 conv, 180
					1 × 1 × 17, softmax	7 × 7 × 180, N + A	7 × 7 × 180, N + A
						7 × 7 × 180, dropout, 50%	7 × 7 × 180, dropout, 30%
						7 × 7 × 180, fully connected	7 × 7 × 180, 3 × 3 conv, 200
						1 × 1 × 17, softmax	7 × 7 × 200, N + A
							7 × 7 × 200, dropout, 50%
							7 × 7 × 200, fully connected
							1 × 1 × 17, softmax

Number after convolutional layer (conv) states the number of filters. N + A + P refers to normalization, activation, and pooling block, and N + A refers to normalization and activation block. Input size is given before each layer or layer block. The number of training parameters (tp) is given before network architectures

Image augmentation using random rotations was applied for the training data before training. Random rotation from − 3° to 3° yielded a final number of 90,288 training sub-images: 4752 sub-images per visible target category and 14,256 sub-images from the non-visible category.

Stochastic gradient descent with momentum solver, constant learning rate 0.01, and momentum 0.9 were used for the training. Cross-entropy loss function was used to calculate loss during training. Mini-batch size was 2736 sub-images, yielding 33 iterations per epoch. CNN1 was trained for 400 epochs and the rest for 300 epochs. The models were validated after each epoch, and the one with the highest validation accuracy was saved. In this study, the multi-class accuracy is defined as the sum of correct classifications over the total number of samples. Finally, statistical analysis against consensus visual scoring was performed on the CNN model with the highest accuracy.

### Statistical analysis

Pairwise comparisons of the observed targets seen by the visual and CNN analyses were made using the Wilcoxon signed-rank test at 5% significance level. Instead of individual specks, the microcalcifications were compared as groups (as shown in Fig. 1D). Fleiss Kappa measure (k) was used to measure the inter-rater agreement between the four reviewers. SPSS statistical software version 25 (IBM Corp., Armonk, NY, USA) was used to perform all statistical tests.

## Results

Table 2 shows the reviewers’ mean scores over the test dataset with standard deviations for fibres, microcalcifications, and masses. The inter-rater agreement between the four reviewers was substantial for fibres (k = 0.69), almost perfect for microcalcifications (k = 0.86), and moderate for masses (k = 0.47).

**Table 2:** Reviewers’ unrounded mean scores over the test dataset and standard deviations for the three target classes

Target	Mean scores per image ± standard deviation			
	Reviewer 1	Reviewer 2	Reviewer 3	Reviewer 4
Fibres	4.2 ± 1.4	4.3 ± 1.4	4.3 ± 1.4	3.5 ± 1.7
Microcalcification groups	3.8 ± 0.7	3.7 ± 0.8	3.7 ± 0.7	3.5 ± 0.9
Masses	4.2 ± 0.9	4.2 ± 0.9	4.2 ± 0.8	3.1 ± 1.0

Test set classification sensitivities and the combined accuracies for the studied CNNs are shown in Table 3. The best overall accuracy (95.2%) of this mammography QC method was achieved with six convolutional layer blocks (CNN4, trained for 246 epochs). CNN5 with seven convolutional blocks was slightly worse in overall accuracy. CNN4 was chosen for further evaluations.

Mean number (± standard deviation) of detected fibres was 4.3 ± 1.4 for visual reviews and 4.4 ± 1.3 for the CNN4 analyses. Wilcoxon signed-rank tests showed no significant difference between the visual reviews and CNN4 results for total fibre scores (p = 0.65). No significant differences were found in pairwise comparison of individual fibres regarding fibre size (0.26 ≤ p ≤ 1.00). CNN4 and the reviewers detected equal number of fibres in 76% of the images. The difference in the number of detected fibres was within ± 1 for 99% of the images.

The mean number of detected microcalcification groups was 3.8 ± 0.8 for both visual reviews and CNN4 analyses. No significant difference emerged between the visual reviews and CNN4 results for the total scores (p = 0.85) or for the pairwise comparison of individual groups with regard to the individual microcalcification size (0.16 ≤ p ≤ 1.00). CNN4 and the reviewers detected equal number of microcalcification groups in 83% of the images. The difference in the number of the detected microcalcification groups was within ± 1 for all the images.

The mean number of detected masses was 4.4 ± 0.9 for visual reviews and 4.5 ± 0.8 for CNN4 analyses. There was no significant difference for total mass scores (p = 0.05). A significant difference was found in pairwise comparison of the smallest individual masses (p = 0.01), whereas pairwise comparison of the larger masses did not reveal significant differences (0.32 ≤ p ≤ 1.00). CNN4 and the reviewers detected equal number of masses in 76% of the images. The difference in the number of detected masses was within ± 1 for 99% of the images.

Table 4 shows the confusion matrix for the CNN4 classifications. There were 17 different categories: 16 categories for different visible targets and one category for non-visible targets. Thus, CNN didn’t just detect the target visibility, but also recognized which target was in question (excluding the ones deemed non-visible). CNN4 classified these targets correctly with a good accuracy, with only small deviations to the nearby categories. However, the least conspicuous targets in each category tended to be more difficult to classify than the larger ones. Classification accuracy for standard QC images was 96.5%. Accuracy for images acquired for the test set with manual exposure or tube voltage settings was 94.0%. For the complete test set, the sensitivity for the non-visible class (equating test set detection specificity if non-visible class is considered the negative class and the combined target classes the positive class) was 87.6%.

**Fig. 4:**
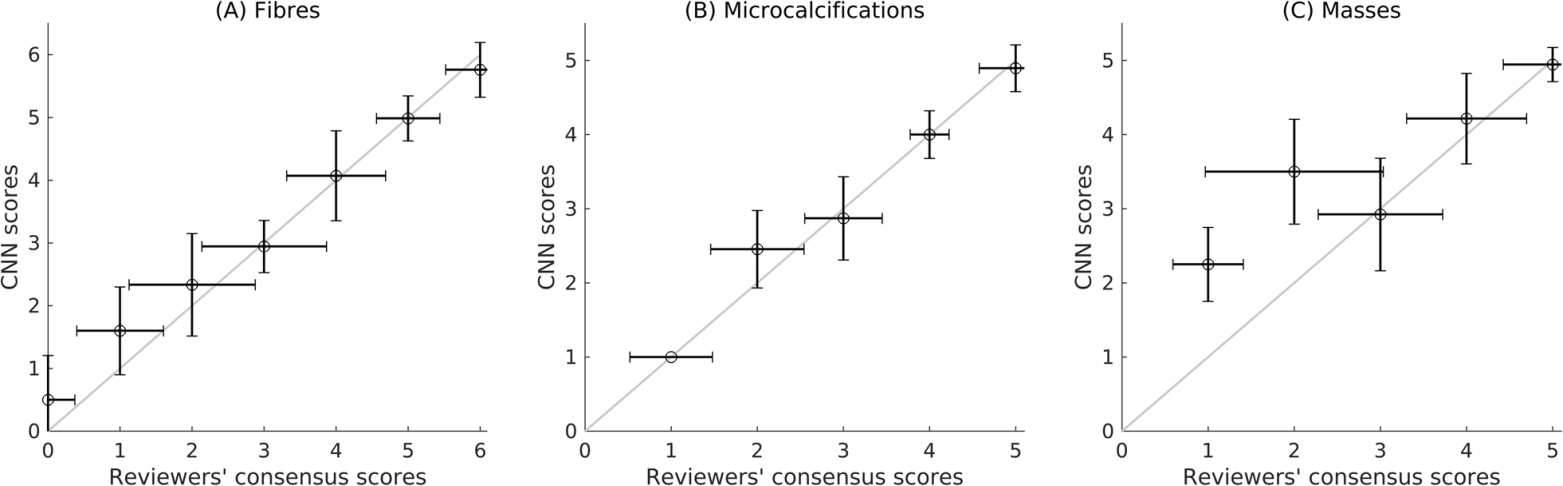
CNN4 total scores (sum of each target type scores) as a function of reviewers’ consensus scores (**A** denotes scores for fibres, **B** scores for microcalcifications, and **C** scores for masses). The error bars represent ± 1 standard deviation for visual and automated scoring. The light grey line represents equal reviewer and CNN scoring

Figure 4 shows the CNN4 target detection performance (total number of detected fibres, microcalcification groups and masses per image) as a function of visual scoring consensus for the whole test dataset. In general, the correspondence was good, and the number of detections increased when image quality increased. The highest differences were observed at the low image quality levels, especially for masses. The CNN4 tended to give higher scores at low image quality levels than the human reviewers. At high image quality levels, the agreement between the CNN4 and the visual review was good. In the tube voltage test set, the CNN4 and visual assessments were in good agreement for the fibres and masses. The CNN detected fewer microcalcification groups in Planmed images at higher voltages, and higher scores at lower voltages in the GE images. However, the tube voltage test set had a limited range in conspicuity in general. In 82% of the false positive predictions by the CNN4 at least one reviewer reported a visible target in the image.

**Table 3:** Target detection sensitivities (including non-visible class) for the eight studied CNNs

	Sensitivity (%)																	
	f1	f2	f3	f4	f5	f6	c1	c2	c3	c4	c5	m1	m2	m3	m4	m5	nonv	Acc
CNN1	100.0	95.9	95.1	92.7	94.8	88.0	99.4	95.6	91.2	94.9	84.2	100.0	94.9	83.8	90.8	96.7	78.3	91.0
CNN2	98.7	96.6	94.4	96.0	93.8	76.0	100.0	97.5	93.9	95.7	84.2	100.0	98.1	89.6	97.9	92.2	85.4	93.6
CNN3	100.0	98.0	97.2	95.2	91.7	72.0	100.0	98.1	96.6	97.4	89.5	100.0	97.4	96.1	99.3	96.7	85.4	94.6
CNN4	100.0	98.6	98.6	96.0	93.8	80.0	100.0	98.7	93.9	98.3	89.5	100.0	98.7	96.1	97.2	94.4	87.6	95.2
CNN5	99.4	98.6	97.2	94.4	93.8	88.0	100.0	100.0	97.3	97.4	89.5	100.0	98.7	96.1	98.6	95.6	85.4	95.0
CNN6	100.0	98.0	95.8	96.0	94.8	80.0	100.0	99.4	93.9	96.6	68.4	100.0	98.7	97.4	97.9	97.8	84.4	94.4
CNN7	100.0	97.3	96.5	93.5	95.8	92.0	100.0	98.7	94.6	94.9	68.4	100.0	99.4	96.8	98.6	96.7	83.8	94.3
CNN8	100.0	99.3	98.6	96.0	97.9	88.0	100.0	99.4	96.6	94.9	73.7	100.0	98.7	95.5	97.2	94.4	82.2	94.2

f stands for fibre (e.g. “f1” means the first, i.e. the most visible, fibre), c stands for microcalcification, m stands for mass, and nonv stands for non-visible target (any class). Acc stands for global accuracy defined as the number of correct classifications over the total number of sub-images. Reviewer consensus is used as the ground truth

**Table 4:** Numbers of detected targets for visual and CNN4 analyses

CNN		Visual																
		f1	f2	f3	f4	f5	f6	c1	c2	c3	c4	c5	m1	m2	m3	m4	m5	nonv
f1		158	0	1	0	0	0	0	0	0	0	0	0	0	0	0	0	2
f2		0	146	0	0	0	0	0	0	0	0	0	0	0	0	0	0	4
f3		0	0	140	0	0	0	0	0	0	0	0	0	0	0	0	0	2
f4		0	0	0	119	0	0	0	0	0	0	0	0	0	0	0	0	2
f5		0	0	0	0	90	0	0	0	0	0	0	0	0	0	0	0	9
f6		0	0	0	1	0	20	0	0	0	0	0	0	0	0	0	0	6
c1		0	0	0	0	0	0	160	0	0	0	0	0	0	0	0	0	0
c2		0	0	0	0	0	0	0	157	0	0	0	0	0	0	0	0	0
c3		0	0	0	0	0	0	0	1	139	0	0	0	0	0	0	0	6
c4		0	0	0	0	0	0	0	0	9	115	0	0	0	0	0	0	3
c5		0	0	0	0	0	0	0	0	0	0	17	0	0	0	0	0	5
m1		0	0	0	0	0	0	0	0	0	0	0	160	0	0	0	0	0
m2		0	0	0	0	0	0	0	0	0	0	0	0	154	1	0	0	4
m3		0	0	0	0	0	0	0	0	0	0	0	0	2	148	4	0	4
m4		0	0	0	0	0	0	0	0	0	0	0	0	0	5	137	1	6
m5		0	0	0	0	0	0	0	0	0	0	0	0	0	0	0	85	17
nonv		0	2	1	4	6	5	0	1	0	2	2	0	0	0	0	4	493

f stands for fibre (e.g. “f1” means the first, i.e. the most visible, fibre), c stands for microcalcification group, m stands for mass, and nonv stands for a non-visible target. The CNN4 was allowed to detect multiple possible targets for each input image patch (therefore, e.g., the sum of the first row is greater than 160)

## Discussion

In this study, eight CNN models were trained and compared against visual assessments for scoring ACR mammography accreditation phantom images. Standard QC images were available from eight mammography systems. A CNN with six convolutional layers yielded the best overall accuracy and resulted in a good agreement with human reviewers suggesting that the method could be utilized in automatic QC. A significant disagreement was found only for the smallest masses. Additionally, CNN tended to give higher scores at low image quality levels than the human reviewers.

In addition to the visibility scoring, the network was trained to identify which target was present in the input image patch. This can act as an internal verification check: one can be more confident of the model’s correct behaviour if the target is both correctly classified as visible as well as correctly recognized (which is already known by the patch location).If the detection of the correct target is ignored, i.e. only considering that some unknown target is visible/non-visible in the input sub-image, the CNN4 accuracy increased from 95 to 96% in the whole test dataset (Table 4).

Kretz et al. have developed a CNN application for mammography QC using the CDMAM phantom [22]. Direct comparison with the current study is not possible due to differences between the phantoms. The CDMAM phantom contains circular structures with varying diameter and thickness, being somewhat simpler than the ACR phantom [22]. However, the current study agrees with the study conclusion that methods based on deep learning can be advantageous for mammography quality assurance.

Visual scoring of QC images is always a subjective task, and confounding factors include prior knowledge of the target location, lighting conditions, experience, and intra-observer variability if the mental model for the scoring criteria changes over time. The inter-rater variability can be seen in the visual scoring results. Reviewer 4 gave substantially lower scores, especially for the fibres and masses, than the other observers. However, Reviewer 4 had a rather small effect on the end results because median was taken between reviewers’ scores. Inter-rater variability was smaller between reviewers 1–3. If Reviewer 4 is ignored on scoring, it mostly has effect on results of the smallest fibres. Viewing conditions and the workstation were the same for all reviewers during the test dataset scoring, although possible differences, e.g. in vigilance and weariness, were not controlled for. The reviewers completed their full task in a relatively short time span without discussing or reviewing borderline cases or disagreements, which may contribute to variation. Despite the difference between the training and test dataset scoring, the CNN model achieved good accuracy. This indicates that the difference between the dataset scorings had only a minor effect. The significant difference for the smallest mass scores might have resulted from the difference in the training and test dataset scoring. The subjectivity and the general difficulty in formulating exact criteria highlight the importance of a consistent and automatic scoring system.

Although the reviewers scored the images at half point accuracy, the scores were rounded to either visible (1 point) or non-visible (0 points). This was primary due to the chosen classification-based CNN architecture. There were only a limited number of half point scores for each target type and therefore classification with half point accuracy was infeasible. This approach might have led to some sensitivity loss as there is always ambiguity on the borderline cases. Additionally, in our approach, the CNN model detected the microcalcification visibility of the complete groups. Individually detected specks could be used to gain higher sensitivity to small changes in performance.

The low-dose images and the smallest targets in every category were the most challenging tasks for the CNN-based automatic scoring. This was also true for the visual assessments. Due to low contrast and high noise contents, it is difficult for the reviewer to score these targets consistently, e.g. discerning the target from the background would be hindered if the location was unknown. A reviewer could even give different results if the same image was scored twice. However, intra-rater variation was not evaluated in this study. Possible inconsistencies in the training data can impact the training process in a negative manner as well as affecting the comparison to the test set. Finding right parameters for CNN training as well as decision threshold is a challenging task. In 82% of the cases CNN falsely predicted visible, at least one reviewer scored the target visible. This indicates a weak signal from the target and underlines the ambiguity of visual evaluations treated as ground truths. Also, in the study design the goal was to mimic human decision-making; image quality overall was reduced to individual and discrete visible/non-visible decision. Alternatively, continuous measures could be used.

Although there were some differences between the reviewer and CNN scores for the low-dose images, a good agreement was achieved. CNN scores followed reviewers’ scores logically when image quality was changed. This is highly important for QC to detect small changes or subtle drifts on the imaging system. When analysing daily QC images, the accuracy at extremely low image quality has limited significance if deterioration from the acceptable level is detected and reported to the user. Radiographer can easily identify drastically worsened image quality immediately after the phantom acquisition.

Exploring different network architectures can improve the model performance. Comparison between different CNN architectures showed that adding more convolutional layers improved accuracy up to six layers. Seven or more convolutional layers decreased the accuracy of non-visible target detection, and thus, overall accuracy. Generalizability to unseen data domain (dose levels) could have been similarly affected. No gain was seen when training different CNNs for each target type. Therefore, a single CNN for all the targets in the image was chosen. Conclusive performance evaluation remains problematic due to the inherent noise (subjectivity) in the ground truth data. Alternatively, unsupervised learning schemes could be applied, e.g. the network could be trained based on a forced A-B testing by presenting it with sub-image pairs with correct target location and with pure noise or background.

In earlier studies, wavelet-based algorithms have been used successfully for image quality scoring of the ACR mammography phantom [28, 29]. For these algorithms, the user must fine-tune thresholds for scoring. This can be difficult, especially when several devices and phantoms are used. For the CNN-based method, this kind of user intervention is not necessary since CNN learns the abstraction and the thresholds autonomously during the training process. This makes CNN-based methods more flexible and adaptable to differences between phantoms. This is important especially in a multi-unit imaging centre environment, where a wide variety of devices and different phantoms may be in use. The benefit of using wavelet-based algorithms is that thresholds can be easily set also for the half points, or barely visible targets. CNN-based methods would likely require a large collection of barely visible targets for accurate training. When comparing CNN to a wavelet-based method [29], CNN gives more accurate results on the target detection task. One of the drawbacks of CNN-based method is that it requires a lot of data for the learning process, and it is time-consuming to score a sufficient amount of phantom images. However, as was demonstrated, it is feasible to use single-observer training data with augmentation and achieve good accuracy.

Study limitations included the number of phantoms and mammography systems, number of reviewers scoring the test images, differences between viewing conditions (and displays), subjectivity of scoring criteria, limited investigation of the data pre-processing and augmentation significance, lack of artefact detection, and limited exploration of different CNN architecture types and hyperparameters. Only eight mammography systems were used for CNN training and testing, and no external testing was performed. Although standardized, each phantom is unique with main differences being the fibre angles and microcalcification patterns. It is possible that the CNN model learned to expect phantom-specific features. Additionally, the physical properties of the ACR phantom limits the absolute sensitivity of the developed QC method. Analysis automation, ideally integrated with the mammography system, would easily allow the use of more complex phantoms with a greater range of targets and target visibilities.

Image rotation was applied in both data pre-processing and image augmentation. This could lead to degraded sharpness for the rotated images, and thus, have a small negative effect on QC performance. However, using rotation to augment images improved the training results in the initial testing, and was chosen for the final models. By the study design, the model training was based on readily available archived QC images with limited variation. The challenges in noise augmentation might have reduced the CNN performance especially at low-dose image classification. The chosen noise augmentation techniques may not fully correspond to that of a real image acquisition. The 10 × 10-pixels patches from specific locations might have been too limited to capture all variability of the noise across a whole image. For example, low frequency noise components are decreased. The NPS for the modified images showed that slight changes in NPS curves can be observed as the patch-based method is not fully congruent to reality. Expanding training and testing sets with greater range of different image qualities or using large homogeneous phantoms used for the noise templates could be used to compensate these limitations. Alternatively, simulated realistic degradation with accurate noise power spectra and artefacts could be used [32].

Computer-aided phantom image analysis could allow wider range of image quality features to be quantified compared to the relatively coarse binary visual evaluation. In this study, the known locations of the phantom targets were utilized, forcing the CNN to focus only on the specific areas of interest. This limits the number of confounding factors that could affect a neural network (e.g. spurious correlations), hopefully improving the performance, but also restricts the obtainable information. One major aspect relevant to mammography QC could be of detecting various image artefacts and degradations (e.g. detector defects, contamination, uniformity, changes in contrast-to-noise ratio), for which the full phantom image could be utilized. CNNs have been previously applied in error detection in other fields [33, 34]. These approaches have the benefit of not necessarily needing a large number of defects or artefacts to be present in the training set, but a large number of normal images from which deviations could be detected. These are easily obtained from archived QC images, after verifying that no artefacts are present. Alternatively, simulated artefacts could be used in model training.

The CNN models in this study were trained using a single reviewer’s scores and the performance was evaluated on the consensus scores. Improving training data quality often translates into improved model performance. Ground truth noise could be mitigated by adding more reviewers or by using more concise scoring criteria and unified viewing conditions (including displays and image windowing). In this study, two different display pairs were used in scoring training/validation dataset and test dataset, which might have a minor effect on the results. This effect is expected to be smaller than the inter- or intra-rater variations. Both display pairs were under quality control for clinical displays and thus considered sufficient for the reading. Scoring criteria could be further unified by more specific instructions or by re-evaluating cases with reviewer disagreement. However, all the reviewers had been working with mammography modality and ACR phantom images scoring. An exact ground truth might, however, not be achievable when visual assessment is considered, and moving towards more objective and quantitative measures might be beneficial. However, the accuracy presented in this study suggest that the developed CNN model is sufficient for daily verification of normal image formation.

## Conclusion

In this study, a novel CNN-based automatic mammography QC phantom scoring system was developed and evaluated. The proposed method was able to score phantom images of different quality in good agreement with human reviewers and can therefore be of benefit in mammography QC.

## Data Availability

The datasets used and/or analysed during the current study are available from the corresponding author on reasonable request.

## Abbreviations

QC: Quality control
CNN: Convolutional neural network
ACR: American College of Radiology
DBT: Digital breast tomosynthesis
DWT: Discrete wavelet transform
AEC: Automatic exposure control
FFDM: Full-field digital mammography
CR: Computed radiography
MGD: Mean glandular dose
NPS: Noise power spectrum
